# Effectiveness of Combined Immunoglobulin and Glucocorticoid Treatments in a Patient With Stiff Limb Syndrome: Case Report and Review of the Literature

**DOI:** 10.3389/fneur.2020.00284

**Published:** 2020-05-08

**Authors:** Juan Huang, Huan-yu Meng, Xian Duan, Wen-wen Li, Hong-Wei Xu, Ya-fang Zhou, Lin Zhou

**Affiliations:** ^1^Department of Geriatric Neurology, Xiangya Hospital, Central South University, Changsha, China; ^2^National Clinical Research Center for Geriatric Disorders, Xiangya Hospital, Central South University, Changsha, China; ^3^Department of Neurology, Ruijin Hospital, School of Medicine, Shanghai Jiao Tong University, Shanghai, China; ^4^Department of Neurology, Hunan Aerospace Hospital, Changsha, China; ^5^Department of Neurology, Xiangya Changde Hospital, Changde, China

**Keywords:** stiff limb syndrome, anti–glutamic acid decarboxylase (anti-GAD) antibody, diazepam, intravenous immunoglobulin, glucocorticoid

## Abstract

Stiff limb syndrome (SLS) is a rare autoimmune-related central nervous system disorder, resulting in stiffness and spasms of limbs since onset with rare involvement of the truncal muscles. However, SLS patients will gain notable effects by appropriate therapy focusing on symptomatic treatment and immunotherapy. We reported on a 55-year-old female who showed typical painful spasms in both lower limbs and abduction of the right eyeball that partially responded to low-dose diazepam and had high-titer anti–glutamic acid decarboxylase (anti-GAD) antibody. Electromyography (EMG) only showed continuous motor unit activity (CMUA) in the anterior tibialis and right triceps. Eventually, our patient was diagnosed with SLS and treated with intravenous immunoglobulin (IVIG) and glucocorticoid combined simultaneously. She obtained notable effects. We also review and summarize the current literature on clinical characteristics, coexisting disease, treatment, and outcome of 40 patients with SLS. We hope that this report will provide a basis for further understanding of SLS and promote the formation of more advanced diagnosis and treatment processes.

## Introduction

Stiff limb syndrome, a variant of stiff-person syndrome (SPS), is a rare autoimmune-related central nervous system disorder (1–3). SLS is characterized by stiffness and spasms limited to the limbs since onset with rare involvement of the truncal muscles. In 1956, Moersch and Woltman reported on 14 patients with fluctuating truncal and limb muscle rigidity and spasms and first defined a newly discovered disease, “stiff man syndrome” (4). Although some progress has been made in the etiology of SLS, the exact mechanism remains controversial. Previous studies claimed that pathogenic autoantibodies impairing γ-aminobutyric acid (GABA) pathways in the brain and spinal cord could be the reason for the clinical manifestations (2). The incidence of SPS is reported to be approximately one in a million (5), while SLS occurs in 13% of SPS patients (6). The prognosis of SLS is variable and largely depends on the underlying autoimmune response, as antibody-positive patients usually have worse clinical outcomes than antibody-negative patients. We recommend that antibody-positive patients receive both long-term immunotherapy and symptomatic treatment, especially for those with chronic symptoms. For antibody-negative patients, symptomatic treatment can be given in the early stage. Whether to give the immunotherapy depends on the severity of symptoms. In this article, we reported on an anti–glutamic acid decarboxylase (anti-GAD) antibody-positive patient with SLS complicating diabetes mellitus (DM). Treatments with intravenous immunoglobulin (IVIG) and glucocorticoid combined simultaneously, instead of sequentially, obtained significant improvement.

## Case Presentation

**Figure 1:**
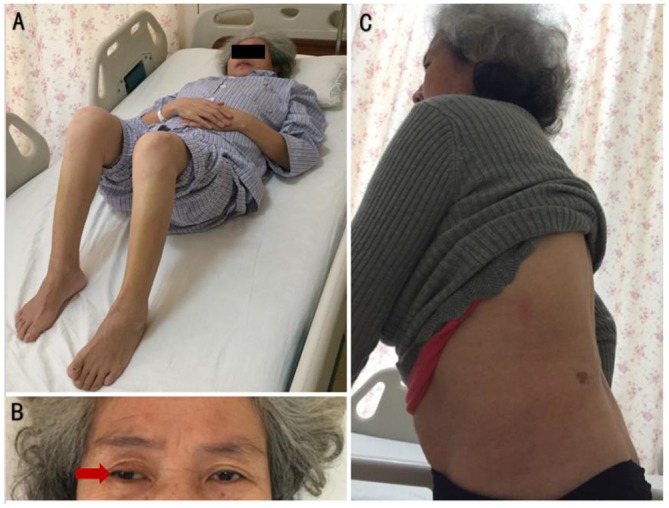
**(A)** Compulsion position. Lower limb flexion due to severe spasms and pains, with painful spasms triggered by slight movements of the lower limbs. **(B)** When gazing forward, the right eyeball (red arrow) was abducted relative to the center of the left eyeball. **(C)** Hyperlordosis of the lumbar spine, without rigidity of the anterior abdominal and lumbar muscles.

A 55-year-old female complained that she had experienced episodic bilateral lower limb spasms and pains since November 2017. In September 2018, she felt intense lower lumbar pain after lifting a heavy weight. Magnetic resonance imaging of the spinal cord demonstrated lumbar hyperlordosis and spinal stenosis. To reduce the compression of the lumbar spinal canal and nerve root canal, the patient underwent a lumbar discectomy + lumbar fusion + internal fixation operation. Although lumbar pain was largely relieved, she noticed that the frequency and duration of lower limb spasms were significantly aggravated. At the third month post-operation, she was bedridden and had to maintain lower limb flexion due to severe spasms and pains (Figure 1A).

Her vital signs were normal. Neurological examinations revealed abduction of the right eyeball when she gazed forward (Figure 1B). In addition, slight lumbar hyperlordosis was found (Figure 1C). Her muscle tone was significantly increased in both lower limbs. Muscle tone was normal in the upper limbs. Deep tendon reflexes were mildly brisk. The Babinski sign was spontaneously positive in both lower limbs. The results from the remainder of the neurological assessments (mental status, cognitive functions, affect, cranial nerves, muscle bulk, and strength sensory examination and coordination) were normal.

**Figure 2:**
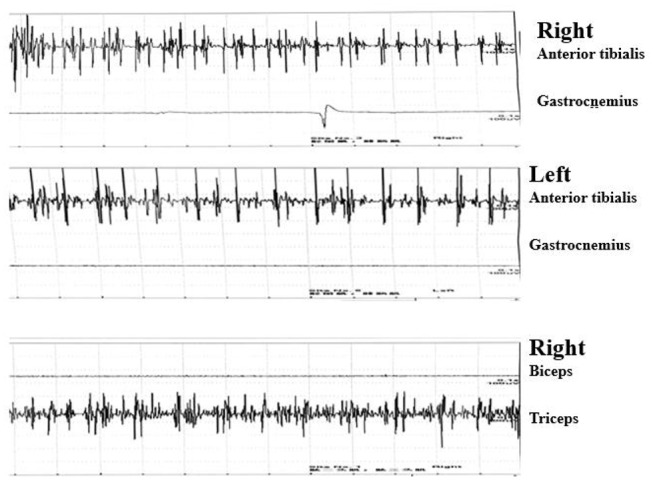
Continuous motor unit activity (CMUA) was found only in the anterior tibialis and right triceps.

Needle electromyography (EMG) revealed continuous motor unit activity (CMUA) only in the anterior tibialis and right triceps (Figure 2). She was found to be positive (++ 1:32) for anti-GAD IgG antibody with an indirect immunofluorescence test (IIFT), strongly positive (+++) for anti-GAD65 IgG antibody by western blot, and negative for anti-amphiphysin IgG antibody (Table 1) with IIFT and western blot. Other laboratory tests after admission showed a moderately increased erythrocyte sedimentation rate [64 mm/h (normal 0–15)] and d-lactate dehydrogenase [288.9 U/L (normal 120–250)], creatine kinase [323.6 U/L (normal 40–200)], and myoglobin levels [141.2 μg/L (normal 0–70)]. Random postprandial blood glucose was up to 13.8mmol/L, and glucose was controlled by daily injections of exogenous insulin. In addition, computed tomography (CT) of the chest, abdomen, pelvis, and brain and 24 h electroencephalography revealed normal results. Laboratory testing results, including a complete blood cell count; a comprehensive metabolic panel including liver, kidney, and thyroid function tests; and tests for levels of electrolytes, C-reactive protein, parathyroid hormone (PTH), HBV-DNA, tumor markers (CEA, AFP, CA15-3, CA125, NSE, CA 72-4, PGI, PGII, and PGR), ANA, ANCA, ACCP, anti-GBM antibody, anti-MPO, anti-PR3, immune factors, rheumatoid factors, and vasculitis factors, were negative. According to the medical history and auxiliary examination results, she was diagnosed with SLS supported by the clinical diagnosis criteria for SPS proposed by Dalakas et al. (2).

**Table 1:** Paraneoplastic neurological antibody results.

**Methods**	**Antibodies**	**Titer**	**Antibodies**	**Titer**
IIFT	Anti-Hu antibody IgG	−	Anti-Yo antibody IgG	−
	Anti-Ri antibody IgG	−	Anti-Ma2 antibody IgG	−
	Anti-CV2 antibody IgG	−	Anti-amphiphysin antibody IgG	−
	Anti-ANNA-3 antibody IgG	−	Anti-Tr antibody IgG	−
	Anti-PCA-2 antibody IgG	−	Anti-GAD antibody IgG	++ 1:32
BLOT	Anti-Tr(DNER) antibody IgG	−	Anti-GAD65 antibody IgG	+++
	Anti-Zic4 antibody IgG	−	Anti-titin antibody IgG	−
	Anti-SOX1 antibody IgG	−	Anti-recoverin antibody IgG	−
	Anti-Hu antibody IgG	−	Anti-Yo antibody IgG	−
	Anti-Ri antibody IgG	−	Anti-PNMA2(Ma2/Ta) antibody IgG	−
	Anti-CV2 antibody IgG	−	Anti-amphiphysin antibody IgG	−

*IIFT, indirect immunofluorescence test*.

Treatment with diazepam 2.5 mg q8h po., tizanidine 2 mg tid po., and oxcarbazepine 150 mg bid po. for 7 days only slightly reduced the frequency of lower limb spasms and pain attacks, but she had to stay bedridden to alleviate her remaining symptoms. Another frustrating fact was that she became drowsy with diazepam dosages >10 mg daily. Thus, immunosuppressive therapy was applied. Subsequently, lower limb spasms and pains rapidly disappeared after IVIG (0.4 g/kg/day for 5 days) simultaneously combined with methylprednisolone sodium succinate for injection (500 mg for 3 days and 250 mg for 3 days, subsequently). After the previously mentioned treatment, she could walk with assistance without painful spasms. Post-treatment levels of d-lactate dehydrogenase, creatine kinase, and myoglobin were normal. After she was discharged from the hospital, the dosage of methylprednisolone tablets 48 mg daily was reduced 4 mg per week until 8 mg daily in the long term. Diazepam was maintained at 2.5 mg q8h po. At 8 months following treatment, she could walk with little assistance without painful spasms.

## Discussion

Although our patient showed typical painful spasms in both lower limbs and abduction of the right eyeball that partially responded to low-dose diazepam and had high-titer anti-GAD antibody, EMG only showed CMUA in the anterior tibialis and right triceps. CMUA was not specific for SPS, as noted in recent literature (7). In addition, there was not enough clinical or neurophysiological evidence of other neurological disorders resulting in pains and spasms. Therefore, according to the clinical manifestations, this patient was diagnosed with SLS (8).

The age, history, sex, affected limbs, laboratory tests, coexisting diseases, treatments, and outcomes of 40 patients with SLS are summarized in Table 2. The median age at diagnosis was 51 years (range, 11–71 years), and the median time from symptom onset to diagnosis was 2.5 years (range, 20 days −11 years). The ratio of males to females was 1:2.3. Stiffness and spasms were usually asymmetric, more often present in one leg than both legs, and rarely involved the upper limbs. SLS presented with increased probabilities of coexisting autoimmune diseases, such as diabetes (15%) and thyroid disease (17.5%), and a close relationship with breast cancer (12.5%), regardless of antibody-positive or antibody-negative status. With the exception of 13 patients having no baseline data, out of 40 total patients, the remainder (27 patients with SLS) included 19 patients (70.4%) who have autoantibodies against inhibitory synaptic proteins (16 with anti-GAD65 antibody, 2 with anti-glycine receptor (anti-GlyR) antibody, 1 with anti-amphiphysin antibody), 2 patients (7.4%) having another autoantibody (anti-islet and anti-axonal antibody), and 6 patients (22.2%) who were seronegative for antibody. Out of 21 antibody-positive patients, 8 patients (38.1%) gained significant improvements by symptomatic treatment and immunotherapy, and 6 patients (28.6%) only using immunotherapy also showed significant improvements. Moreover, out of six antibody-negative patients, five patients (83.3%) gained significant improvements after symptomatic treatment. We can infer further that the majority (14/21, 66.7%) of antibody-positive patients gained significant improvements by immunotherapy, while most antibody-negative patients (5/6, 83.3%) showed significant improvements with symptomatic treatment. We recommend that antibody-positive patients receive both long-term immunotherapy and symptomatic treatment, especially for those with chronic symptoms. For antibody-negative patients, symptomatic treatment can be given in the early stage. Whether to give the immunotherapy depends on the severity of symptoms.

**Table 2:** Reported cases of stiff limb syndrome.

**References**	**Age**	**History**	**Sex**	**Affected limbs**	**Laboratory tests**	**Coexisting disease**	**Treatment**	**Outcome**
1. Saiz et al. (9)	63	/	F	Right leg	Anti-GAD antibody (+)	Hyperthyroidism	Diazepam, IVIG (0.4 g/kg/d for 5 days)	Improved significantly
2. Saiz et al. (9)	56	11 y	F	Right leg	Anti-GAD antibody (+)	Epilepsy and DM	Benzodiazepines	Progressed slowly
3. Barker et al. (8)	41	6.2 y (1–19 y)	8 F 5 M	Leg>arm	2/13 Anti-GAD antibody (+), 5/13 other autoantibody (+)	0/13 DM	Diazepam, baclofen, and steroids	7 improved partially with diazepam or/and baclofen, 2 did not respond to diazepam and/or baclofen, 1 improved partially with steroids, and 4 did not respond to steroids
4. Silverman (10)	68	1 m	F	Both legs	Anti-GAD antibody (+)	Breast cancer and Graves' disease	Clonazepam and a trial of oral dexamethasone	Death
5. Souza-Lima et al. (11)	60	4 y	F	Left leg	Anti-GAD antibody (+)	/	Two cycles of IVIG (0.4 g/kg/d for 5 days)	Improved significantly
6. Gurol et al. (12)	28	5 y	F	Right leg	Anti-GAD antibody (+)	DM and hyperthyroidism	Clonazepam (4 mg/d)	Improved significantly
7. Coles and Barker (13)	50	10 y	F	Face, trunk, and limbs	Anti-GAD antibody (–) and anti-axonal antibody (+)	Viral meningitis and seronegative polyarthritis	Diazepam and baclofen, intravenous methylprednisolone, IVIG, four courses of plasma exchange, and oral cyclophosphamide	Improved significantly
8. Schiff et al. (14)	47	9 m	F	Right foot, ankles, and toes	Anti-GAD65 antibody (+) and anti-islet cell antibody (+)	Multiple myeloma, hypothyroidism, and focal epilepsy	Diazepam (2.5 mg bid)	Improved significantly
9. Hongyuan et al. (15)	45	40 d	F	Both limbs	Islet cytoplasmic autoantibody (+) and anti-GAD antibody (–)	Breast cancer	Benzodiazepines (40–60 mg/d), intravenous methylprednisolone (500 mg/d for 6 days), oral prednisolone (60 mg/d for 2 month), and IVIG sequentially	Improved significantly
10. Bartsch et al. (16)	69	5 y	F	Right leg	Anti-GAD antibody (+)	Normal	Diazepam (5 mg/d)	Improved significantly
11. Weatherby et al. (17)	41	4 y	F	Both legs	Anti-GAD antibody (–)	Delivered a healthy baby	Diazepam (15–60 mg/d) and baclofen (25–100 mg/d)	Improved significantly
12. Holmoy (18)	39	9 y	F	Right leg	Anti-GAD antibody (+)	Hypothyroidism	Diazepam (5–7 mg/d) and gabapentin (2,400 mg/d)	Asymptomatic
13. Dubow and Zadikoff (19)	52	1 m	F	Right leg	Anti-GAD antibody (–) and anti-amphiphysin antibody (–)	Breast cancer and navicular stress fracture	Diazepam (40 mg/d)	Improved significantly
14. Teive et al. (20)	11	2 y	F	Right upper and both lower limbs	Anti-GAD antibody (+)	Upper respiratory tract viral infection	IVIG (initial dose: 0.4 g/kg/d for 5 days, followed by monthly doses of 0.4 g/kg for 1 day)	Improved significantly
15. Misra et al. (21)	42	20 d	M	Both legs	Anti-GAD antibody (–)	Hiccups and vomiting	Diazepam (37.5 mg/d)	Asymptomatic
16. Ughratdar et al. (22)	44	6 y	M	Right leg	Anti-GAD antibody (–)	Back injury	Benzodiazepines, baclofen, various opioid medications, and implantable pulse generator	Improved significantly
17. Hajjioui et al. (23)	49	2 y	M	Both legs	Anti-GAD antibody (–)	Normal	Diazepam (25 mg/d) and baclofen (30 mg/d)	Improved significantly
18. Agarwal and Ichaporia (24)	55	2 m	F	Right leg and foot	Anti-GAD antibody (+)	Breast cancer	Diazepam (10–60 mg/d), baclofen (20–30 mg/d), and prednisolone (10–60 mg/d)	Improved significantly
19. Iwata et al. (25)	29	2 y	M	Both legs	Anti-GAD antibody (–) and anti-amphiphysin antibody (–)	/	Diazepam (20 mg/d)	Improved significantly
20. Castelnovo et al. (26)	63	3 y	F	Left leg	Anti-GAD antibody (+)	DM and pernicious anemia	Diazepam (10 mg/d) and IVIG monthly	Improved significantly
21. Chamard et al. (27)	65	2 w	F	Both legs	Anti-GAD antibody (–) and anti-amphiphysin antibody (+)	Breast cancer, DM, thyroid goiter, paraneoplastic transverse myelitis	Intravenous glucocorticoid (1 g/d for 5 days), diazepam (15 mg/d), baclofen (10 mg tid), and tizanidine	Improved significantly
22. Anagnostou and Zambelis (28)	40	9 y	F	Both legs	Anti-GAD antibody (+)	Normal	Diazepam (10 mg bid) and botulinum toxin A (900 U)	Improved significantly
23. Derksen et al. (29)	61	4 w	M	Both legs	Anti-GlyR antibody (+), anti-GAD antibody (–), and anti-amphiphysin antibody (–)	Chronic lymphocytic leukemia	Baclofen, clonazepam, IVIG, five cycles of PE, bendamustine, and two cycles of rituximab	Improved significantly
24. Balint et al. (30)	69	/	M	Right leg	Anti-GAD antibody (+)	DM	Diazepam, tizanidine corticosteroid pulse, and oral tapering	Controlled
25. Balint et al. (30)	71	/	M	Left leg	Anti-GAD antibody (+)	/	IVIG, pulse prednisone, followed by weaning doses of oral prednisone	Controlled
26. Duwicquet et al. (31)	69	4 y	F	Right leg	Anti-GAD antibody (+)	Hypothyroidism	IVIG (0.4 g/kg/d for 5 days) every month over 6 months	Improved partially
27. Maeda et al. (32)	48	5 y	F	Both legs	Anti-GlyR antibody (+)	/	Intravenous methylprednisolone (500 mg/d, 3 days) and oral prednisolone (30 mg/d)	Asymptomatic
28. Present report report (2019)	55	2 y	F	Both legs	Anti-GAD antibody (+)	DM and back injury	Diazepam (7.5 mg/d), tizanidine (6 mg/d), IVIG (0.4 g/kg/d for 5 days), glucocorticoid (500 mg for 3 days and 250 mg for 3 days, subsequently), and oral tapering	Improved significantly

*y, year; m, month; d, day; F, female; M, male; anti-GAD antibody, anti–glutamic acid decarboxylase antibody; anti-GlyR antibody, anti-glycine receptor antibody; IVIG, intravenous immunoglobulin; PE, plasma exchange; DM, diabetes mellitus*.

In 1988, Solimena et al. first reported autoantibody in serum to human brain GAD (anti-GAD) in a patient with SPS, epilepsy, and type I DM (33). Anti-GAD antibody exists in diseases such as type I DM, SPS, cerebellar ataxia, and other neurological disorders (epilepsy, idiopathic limbic encephalitis, and myasthenia gravis) (34). GAD is a pyridoxal 59-phosphate-dependent enzyme that converts glutamate into GABA (35–37). GABA acts as a major inhibitory neurotransmitter in the brain and spinal cord by triggering chloride channels to open, causing the inward movement of chloride into the cell, resulting in membrane hyperpolarization. Anti-GAD antibodies are produced intrathecally to inhibit GAD bioactivity and downregulate GABA expression, resulting in low level of GABA in the brain and cerebrospinal fluid and eventually leading to various clinical manifestations (36).

The treatment of SLS focuses on symptomatic treatment and immunotherapy. Diazepam could increase the opening frequency of the GABA_A_ receptor and lead to hyperpolarization to inhibit excessive neurophysiological activities so that the rigidity and spasms of muscles could be relieved (38, 39). The effective dose ranges from 5 to 60 mg/day for patients with SLS. Most patients with SLS respond to diazepam, but many of them cannot tolerate the high-dose-related adverse effects, including respiratory depression, drowsiness, and dysarthria. The adverse effects of high-dose diazepam have become a major reason why symptomatic treatment fails. Our patient's muscle spasms were partially reduced but were not eliminated due to high doses of diazepam causing drowsiness. The combination of diazepam with other symptomatic treatment, such as baclofen (20–100 mg), clonazepam (4–10 mg/day), tizanidine (6–36 mg/day), and gabapentin (up to 2,400 mg/day), has also been used for relieving SLS symptoms (12, 16–18, 24, 29, 30).

When patients with SLS incompletely respond to diazepam and/or baclofen, IVIG (2 g/kg in 2–5 days) is recommended to improve significant disability in daily activities and is a safe and effective therapy for patients with SLS (9, 20, 30, 31). The effects of IVIG usually last several months, and another IVIG session is started with the return of symptoms (11, 20, 26, 31). Immunoglobulin may suppress the activities of anti-GAD65 antibody by accelerating the rate of IgG catabolism, and the other immunomodulatory effects of immunoglobulin on the neutralization of cytokines and T cells may also play a complementary role (7, 40). Glucocorticoids (e.g., induced as pulse therapy with 500–1,000 mg/day methylprednisolone for 3–5 days and an oral maintenance dose with prednisone 1 mg/kg or 60–80 mg po., oral tapering) could be efficient in treating SLS (24, 27, 30, 32). Plasma exchange may benefit patients with SLS who failed to respond to baclofen and diazepam and cannot tolerate IVIG and glucocorticoids by reducing serum levels of antibodies and other proinflammatory mediators (13, 29). The use of “further immunotherapy” (rituximab, oral cyclophosphamide) is rare in SLS. Interestingly, oral cyclophosphamide was effective in a patient with SLS who was reluctant to accept repeated courses of plasma exchange (13). Another patient with SLS used rituximab and bendamustine (first day, rituximab 350 mg/m^2^; second and third days, bendamustine 90 mg/m^2^) to deplete mature B cells, which caused deadly side effects (mydriasis, tachycardia, high blood pressure, hyperthermia, and even cardiac arrest) (29). Therefore, caution should be taken when starting “further immunotherapy” due to a high risk of adverse events, such as severe autonomic instability, opportunistic infections, and neoplasms.

## Conclusion

SLS is initially limited to stiffness and spasms of the limbs. Many SLS patients ultimately progress to whole-body stiffness and spasms without appropriate treatment. The etiology in most SLS cases is an autoimmune process, and in rare cases coexisting malignant tumors, especially breast tumors. The treatment of SLS follows the principle of symptomatic treatment and immunotherapy. We recommend that antibody-positive patients receive both long-term immunotherapy and symptomatic treatment, especially for those with chronic symptoms. For antibody-negative patients, symptomatic treatment can be given in the early stage. Whether to give the immunotherapy depends on the severity of symptoms. In summary, we reported on an SLS patient with elevated anti-GAD antibody in serum. Our patient gained notable effects by treating with IVIG and glucocorticoid combined simultaneously. As a result, she resumed normal daily activities after the treatment. However, our patient should maintain long-term low-dose methylprednisolone and diazepam, and tumor monitoring is still necessary. We hope our article provides further understanding of SLS and becomes a reference for future treatment.

## Data Availability Statement

All datasets generated for this study are included in the article/supplementary material.

## Ethics Statement

Written informed consent was obtained from the individual(s) for the publication of any potentially identifiable images or data included in this article.

## Author Contributions

JH and HM participated in writing of the paper. XD, WL and H-WX participated in collecting the information of the paper. YZ participated in the clinical data analysis. LZ participated in the revising of the paper.

## Conflict of Interest

The authors declare that the research was conducted in the absence of any commercial or financial relationships that could be construed as a potential conflict of interest.
